# RGD Forever!—Past, Present, and Future of a 3-Letter-Code in Radiopharmacy and Life Sciences

**DOI:** 10.3390/ph16010056

**Published:** 2022-12-30

**Authors:** Johannes Notni

**Affiliations:** 1 Institut Für Pathologie und Pathologische Anatomie, School of Medicine, Technische Universität München, Trogerstr. 18, 81675 München, Germany; tum@notni.de; Tel.: +49-89-4140-6075; Fax: +49-89-4140-6949; 2 TRIMT GmbH, 01454 Radeberg, Germany

**Keywords:** integrins, peptides, molecular imaging, radionuclide therapy, targeted delivery

## Abstract

“RGD” is frequently pictured as a ligand for αvβ3-integrin and useful for molecular targeting of angiogenesis—which is about as simplistic as the idea that laser beams are green or red and particularly useful for arming spaceships. There is, however, much more to RGD. In particular, targeting angiogenesis is likely not the most significant stronghold of RGD-comprising constructs. RGD is the one-letter code of a very short peptide sequence, arginine-lysine-aspartate, which is recognized by eight different integrins, namely, α(IIb)β3, α5β1, α8β1, and the five dimers that αv forms with β1, β3, β5, β6, and β8. These 8 RGD receptors form an own subset among the entire class of 24 known integrins, which furthermore comprises another three distinct groups (4 collagen receptors, 4 laminin receptors, and 8 leukocyte receptors). However, the 8 RGD-recognizing integrins are far from being alike. They do not even share the same tissue prevalences and functions, but are expressed on fundamentally different cell types and fulfill the most diverse biological tasks. For example, α(IIb)β3 is found on platelets and mediates thrombus formation, whereas αvβ6- and αvβ8-integrin are expressed on epithelial cells, activate TFG-β, and thus may promote cancer progression and invasion as well as fibrosis. Recent non-clinical experiments and clinical findings suggest that the highly specific expression of αvβ6-integrin by some carcinoma types, in combination with the availability of the corresponding small-molecule ligands, may open a multitude of new and promising avenues for improved cancer diagnosis and therapy, including, but not limited to, radiopharmaceutical approaches.

## 1. Prologue: About Ideas and Their Public Perception

After Theodore H. Maiman had completed the construction of a device that produced a beam of highly intense, coherent visible light by stimulated emission from an externally energized ruby rod—the first LASER—he attempted to publish his invention in Physical Review Letters but got rejected twice, for the reason of insignificance (the paper finally ended up “just” in *Nature* as a timeless classic of less than 300 words) [1]. Admittedly, it must have been impossible in 1960 to anticipate that the “laser” (once coined as an acronym, but nowadays written in lowercase letters and used both as a noun and a verb) would become indispensable for virtually all technologies and industry products of the 21st century. Lasers, however, captured people’s fantasy early on. Even a completely imaginary idea, such as their use in interstellar warfare, which was shaped by a highly successful movie series, still persists in the general public’s mind. This story illustrates that a widely held idea or an earlier prognosis do not necessarily reflect the current focus or future direction of a particular field (notwithstanding the troubling prospect that spaceships firing laser beams may be part of our future after all). Admittedly, the term “RGD” is far less ingrained in the collective consciousness of mankind than the word “laser”. One similarity, however, is that there is apparently a difference between what many researchers associate with “RGD” and what else it actually implicates.

## 2. The Origins and Perception of RGD

The fundamental idea that ignited RGD’s flight to fame can be traced back to its origins fairly easily. Pierschbacher and Ruoslahti discovered in 1984 that some integrins (a class of 24 heterodimeric cell adhesion receptors [2]) bind to their native ligands, large extracellular matrix (ECM) proteins, by recognition of the short sequence arginine-glycine-aspartate (abbreviated RGD in the one-letter code) which repeatedly occurs in these proteins [3]. Shortly thereafter, in 1991, Kessler and coworkers designed a small, cyclic peptide motif containing the RGD sequence for antagonizing integrin-mediated cell-binding to certain ECM proteins [4]. This class of cyclic pentapeptides, for example, c(RGDfK) or c(RGDyK), later turned out to be an ideal targeting motif for life science applications [5]. It is not metabolized or degraded in vivo, easy to synthesize by standard methods, and binds with high selectivity to one particular integrin, αvβ3 [6]. The puzzle was completed by Brooks, Clark, and Cheresh in 1994, who discovered that the vascular αvβ3-integrin is required for angiogenesis (sometimes termed neo-vascularization) [7]. This key process, referring to the sprouting of new blood or lymphatic vessels from existing ones, is not only widely encountered in developmental biology and wound healing [8]. Angiogenesis is furthermore a critical step in the development of solid tumors which, in order to grow beyond the size of a few millimeters, must secure their supply of nutrients and oxygen by establishing a vessel system. The corresponding, necessary change in the tumor cells’ signaling behavior, leading to the recruitment and activation of endothelial cells, is widely referred to as “angiogenic switch” [9], and frequently involves overexpression of αvβ3-integrin.

The life science community thus came into possession of an intriguingly straightforward concept and the appropriate molecular tools to target early development stages of tumors in general, with the vision to exploit αvβ3-integrin antagonization for therapeutic and diagnostic approaches [10,11,12]. The rise of “RGD peptides” was further catalyzed by the fact that no protection of intellectual property permanently hindered their widespread use, and that the terminal amino group of the lysine side chain in the c(RGDxK) motif may be functionalized without severely compromising target affinity or -selectivity [6]. This feature made conjugation or surface grafting an easy endeavor, paving the road toward all sorts of αvβ3-integrin homing agents, such as fluorescence markers, radiopharmaceuticals, drug conjugates, nanoparticles, micelles, and other constructs [13].

“RGD peptides” and the associated narrative of targeting angiogenesis thus became a very popular concept in life sciences. As of late 2022, a search for the term “RDG” in Clarivate’s Web of Science^™^ database yielded more than 15,000 hits, more than half of which (approx. 7,800) were published within the past decade, reaching an all-time peak of 742 in 2016. One consequence of this ubiquity is that it is practically impossible for a single person to digest all this information (unless one has been willing to read 2 RGD papers every single day for the last 10–15 years). A quick glance, however, suggests that RGD applications related to angiogenesis and αvβ3-integrin are somewhat overrepresented. At least in the field of radiopharmacy, an overwhelmingly large percentage of all “RGD”-related publications deal with αvβ3-integrin, angiogenesis, or neo-vascularization. This is where a mechanism familiar from news media and social networks comes into play, most succinctly expressed in the adage “vox populi, vox dei”. The constant repetition of one and the same idea by many can create an illusion of a higher level of evidence or relevance than actually exists and thus, drive related developments even if they gradually become less relevant for future research.

## 3. The History of the RGD Motif in (Radio-)pharmaceuticals

Let us leave that aside for now and have a look at how it all started. The first αvβ3-integrin homing radiotracers have been developed more than two decades ago [14]. Positron emission tomography (PET) imaging in humans showed their ability to visualize different tumor types [15], which resulted in a genuine excitement in the research community at the prospect of being able to image such a fundamental process like angiogenesis [16]. Radiolabeled αvβ3-integrin ligands thus were thought to be a key asset, and many different ones were developed [17] and even transferred into clinical studies [18]. None of the clinical candidates, however, has gained traction in public healthcare because a clinical added value could not yet be convincingly demonstrated [19]. This is not due to a general reluctance of medical practitioners or the pharmaceutical industry to embrace radiopharmaceuticals. On the contrary, these players have recently succeeded in integrating sst2- and PSMA-addressing agents such as NETSPOT^®^, Lutathera^®^, or Pluvicto^®^ into healthcare and reimbursement schemes. The difference is found in the targets, though. In case of of αvβ3-integrin, the main obstacle may have consisted in a substantial expression in various healthy tissues. At least, this is suggested by a typical background signal seen in images of healthy volunteers and patients acquired with variety of αvβ3-tracers [20]. The quality of the available radiotracers is apparently not a problem, but the target itself whose potential is further limited by the lack of specificity of the signal for the targeted biological mechanism, angiogenesis. Hotspots in images cannot be clearly assigned to enhanced angiogenic activity because αvβ3-integrin is also expressed by some tumor cell lines (a fact that led to the apparently widespread but incorrect assumption that RGD is generally a tumor targeting motif)—all the more because there is no strict causal relationship between angiogenesis and αvβ3-integrin expression [21]. The quite straightforward concept derived from the above-mentioned early studies, i.e., that αvβ3-integrin expression is a proxy for angiogenic activity [7], has been revised significantly in the meantime. Collected experimental evidence led to the insight that the abundance of αvβ3-integrin is most likely not quantitatively correlated with the actual extent of angiogenesis in a given tissue [22]. Against this background, some studies suggested that radiotracers targeting another RGD-binding integrin, α5β1, might be more suitable for imaging of angiogenesis [23,24], but definitive clinical proof is still lacking. In addition, extensive development of tracers for molecular imaging of other αv-integrins, such as αvβ8 [25,26] and particularly αvβ6 [27,28,29,30], has been carried out over the past 15 years, but seems to have been somehow obscured by a continuous stream of publications dealing with probes for αvβ3-integrin.

The focus on the subtype αvβ3 was apparently less pronounced in some other research areas. As far as medicinal chemistry and bioligand design are concerned, development has never stopped at the stage of cyclic RGD pentapeptides anyway, nor has it ever been limited to αvβ3-integrin. This is because over the years, the RGD sequence was shown to be the natural ligand recognition motif of eight different integrins [31]. These are α(IIb)β3, α5β1, and α8β1, and furthermore all 5 αv integrins (αvβ1, αvβ3, αvβ5, αvβ6, and αvβ8), which is why RGD is sometimes referred to as a ligand for targeting αv integrins (which, as a solitary statement, is actually not completely wrong but too imprecise to be considered correct). In other words, nature has managed to use one and the same tiny peptide sequence to enable a more or less selective recognition of large proteins by eight different integrins. Thus, it quickly became clear that the RGD sequence apparently needed to be incorporated into tailored larger molecules or scaffolds to achieve selectivity. The goal was to limit the structural flexibility or freedom of movement, so that the conformationally fixed motif would only fit into the RGD binding pocket of one of the integrins [6]. In addition, there have been successful strategies to selectively target individual RGD-binding integrins with molecules not comprising the RGD sequence, for example, peptides comprising other amino acid sequences like RTD for targeting αvβ6, peptidomimetic motifs like *iso*DGR for targeting α5β1, or entirely non-peptidic structures like the anti-platelet drug tirofiban that binds to α(IIb)β3-integrin. These approaches, which are part of a multitude of selective ligands for RGD-binding integrins and a wealth of pertinent research, have been comprehensively reviewed previously [5,12,13], which will thus not be repeated here. It remains to be asked, however, why this hidden treasure has apparently only been partially unearthed so far. Regardless of what the answer to that question might be, it seems like a good idea to get fully started right now [32].

## 4. RDG-Binding Integrins as Targets for Radio-Theranostics

As outlined above, radiolabeled ligands for imaging of αvβ3-integrin have been the first stronghold of RGD in radiopharmacy but never ended up in clinical routine. It might thus worth having a closer look on the possible reasons and their implications for future research. It is first useful to recall that the clinical success of both classes of targeted radiopharmaceuticals, i.e., imaging agents and targeted therapeutics, is much more determined by target-mediated, yet undesirable, accumulation in healthy organs and tissues than is the case with normal drugs.

The mode of action of most standard medications relies on the interaction with, or blockade of, a certain receptor or mechanism. The drug is expediently administered in sufficiently large amounts to saturate as many target sites as possible, above all, those which convey the desired therapeutic effect. Other available targets in healthy tissue are of course also engaged, which is usually unavoidable but acceptable unless it results in an intolerable toxic effect. In contrast, radioactive imaging probes are administered in amounts several (usually 4–5) orders of magnitude lower, resulting in virtually negligible tissue concentrations where only a small percentage of available target sites in the body, whether in healthy or diseased tissue, is engaged at all. It is obvious that in this case, target engagement and tracer accumulation in non-diseased tissue play a crucial role, as this undermines the intended purpose of a radiotracer, which is to accumulate as selectively as possible in diseased tissue to allow unambiguous interpretation of imaging data.

This leads directly to the requirement that a suitable target for radiopharmaceuticals should be as disease-specific as possible, i.e., its presence should be restricted, as far as possible, exclusively to diseased sites. This imperative applies all the more to radiotherapeutics, that is, radiopharmaceuticals which comprise particle emitters and whose purpose is to engage target cells and kill them by irradiation at close range. Off-target accumulation, such as non-disease specific or physiological organ uptake, is more and more critically viewed during the early, preclinical investigation of radio-theranostics for cancer therapy because of the risk of facing problems with radiation-related toxicity at a later stage of clinical development.

**Figure 1:**
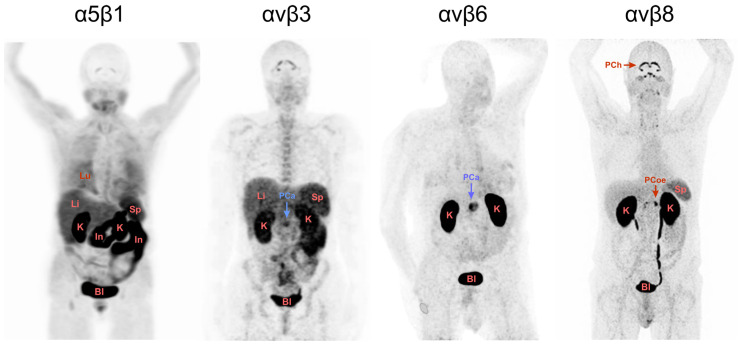
PET images (maximum intensity projections, MIP, seen from ventral) in human subjects, acquired 70–90 min after administration of 100–150 MBq of ^68^Ga-labeled radiopharmaceuticals selective for the integrin subtypes α5β1 (^68^Ga-Aquibeprin) [23], αvβ3 (^68^Ga-Avebetrin) [33], αvβ6 (^68^Ga-Trivehexin) [30], and αvβ8 (^68^Ga-Triveoctin) [26]. Two subjects had a pancreatic carcinoma in the pancreatic head (indicated by blue arrows labeled PCa). Renal excretion of tracers resulted in strong kidney signals (labeled K). Other uptakes are observed in lungs (Lu), liver (Li), intestines (In), spleen (Sp), urinary bladder (Bl), choroid plexus (PCh) and celiac plexus (PCoe). Copyright notice: Parts of the figure were adapted from *Eur. J. Nucl. Med. Mol. Imaging* **2021**, *48*, 4107–4108 (αvβ6 PET) and *EJNMMI Res.* **2020**, *10*, 133 (αvβ8 PET) under Creative Commons CC BY 4.0. Red arrows: Non-cancer related uptakes; Blue arrows: Known cancer lesions.

A look at some of our recent data is helpful in illustrating these relations and their implications for RGD. We previously developed a series of structurally related PET probes for four different RGD-binding integrins, ^68^Ga-Aquibeprin and ^68^Ga-Avebetrin for α5β1 and αvβ3, respectively [23,24]; ^68^Ga-Trivehexin for αvβ6 [30]; and ^68^Ga-Triveoctin for αvβ8 [26]. They all have been designed as homo-trimers of the respective peptidic or peptidomimetic integrin ligands in order to achieve a higher avidity and, thus, an increased target-specific uptake [22]. Since all four agents have been described in detail before and the following considerations are largely independent on the actual structure or molecular design of the probes, these aspects are not further discussed here. It is however important to mention that based on the preclinical data, a high target specificity can be assumed for all four tracers, suggesting that any observed organ or tissue accumulation is predominantly caused by actual integrin expression in the respective compartments. An exception are the kidneys, where a strong signal due to renal excretion is observed for all probes, an artifact that occurs with most established PET imaging agents. Figure 1 shows maximum intensity projections (MIPs) of PET scans acquired under comparable conditions using the four tracers, illustrating the fundamentally different suitability of the four regarded RGD-binding integrins as targets for molecular medicine.

To begin with the most popular integrin αvβ3, the respective PET image in Figure 1 closely resembles those obtained with other αvβ3-tracers and exhibits the typical and well-known physiological uptake pattern [20]. Apart from the expected excretion-related signals in kidney and urinary bladder, uptakes caused by physiological expression are observed in the spleen, liver, and intestines, limiting the potential diagnostic capabilities of this class of radiopharmaceuticals as well as their utility for radiotherapy.

A similar situation is found for the α5β1-integrin PET. Figure 1 shows a distinct pattern of strong organ uptakes, likely limiting the prospects for diagnostic utility. However, the image shows the PET scan of a patient originally scheduled for diagnosis of myocardial infarction, who had several comorbidities, presumably including inflammatory bowel disease, which could be responsible for the observed intestinal uptake. Since there are no further images in the literature that have been acquired using comparable tracers, it is not easy to draw final conclusions at that time. This situation urgently calls for future investigations to consolidate the view on α5β1-integrin imaging, which is nonetheless most likely a promising target for imaging and therapy of cancer [34] and other diseases [35].

The αvβ8-integrin PET in Figure 1 furthermore suggests that the physiological expression of αvβ8 in most organs and tissues is low, which is in good agreement with the literature and encourages further exploration as a theranostic target [36]. Although the origin of the pronounced uptake in the choroid and celiac plexus is still unclear and deserves further investigation, αvβ8-integrin apparently fulfils the above-mentioned requirement of a low background expression. However, the αvβ8-integrin PET shown is the only one of its kind. Apart from this image of a healthy subject, no other in vivo images of αvβ8-integrin expression in humans were reported so far, which again limits the validity of the conclusion drawn.

The αvβ6-integrin PET, however, leaves little doubt that this RGD-recognizing integrin is a very promising molecular target indeed, particularly in the context of oncology. The image shows no significant uptake in any organ except the excretion-related ones in the kidneys and urinary bladder, which is consistent with a large body of histological data showing that αvβ6-integrin is only weakly expressed in adult human tissues [12]. Moreover, the αvβ6-PET in Figure 1 actually shows a patient with pancreatic ductal adenocarcinoma (PDAC), wherein the cancer lesion exhibits an intense tracer uptake. It can be assumed that a clinical use for the respective tracer (Ga-68-Trivehexin [30]) in cancer diagnostics will emerge, especially because a solid body of PET/CT data has already been generated using this tracer for patients with PDAC and other cancers, confirming this view [37]. Furthermore, one can not fail to notice the striking difference to the adjacent αvβ3-integrin PET which also shows a patient with PDAC. Here, the background almost obscures the lesion and may lead to ambiguous results regarding the possible detection of metastases.

The currently available evidence thus suggests that other integrins than αvβ3, most likely, αvβ6, will shape the future of radio-theranostics targeting RGD-recognizing integrins [21,22]. This applies all the more because αvβ6 is not only virtually absent in healthy human tissues, but is also reportedly highly expressed on the surface of various cancer cell types [38], most notably PDAC [39,40], whose poor survival rates urgently call for more effective therapies. It is recognized that any future radio-therapeutic should be characterized by a low kidney uptake to avoid renal damage, but this does not detract from the apparent extraordinary potential of αvβ6-integrin as a theranostic target.

At this point, it is worth noticing that not just one, but several substantially different classes of peptidic αvβ6-integrin ligands have been described so far, which have been summarized in recent reviews [12,13]. They include, but are not limited to, (a) the linear A20FMDV peptide, originally developed by Marshall and colleagues [41] and later converted into a clinical PET tracer by Sutcliffe and coworkers [28]; (b) cystein-knot peptides, first reported by Kimura et al. [42] and used for imaging of cancer and fibrosis in humans [29]; (c) two cyclic peptides, SFITGv6 and SFLAP3, engineered at the DKFZ Heidelberg on the basis of the sunflower trypsin inhibitor scaffold and tested for cancer imaging in patients [27]; and (d) a class of cyclic nonapeptides developed by Kessler and colleagues [43], which was further modified and applied for clinical PET imaging of carcinomas a few years later [30]. All of these ligand motifs comprise the RGD sequence and exhibit high affinity and selectivity for αvβ6-integrin. There seem to be many ways to teach an old dog new tricks, i.e., to turn the venerable RGD into novel and promising tools for molecular medicine. This process is apparently not about to come to an end, but on the contrary, seems to be accelerating and diversifying. To stay with the topic, novel radiolabelled αvβ6-integrin binding peptides comprising the RGD sequence continue to emerge, such as the linear 21-mer 5G recently reported by Sutcliffe and colleagues [44], the cyclic octapeptide cycratide introduced by Wang and coworkers [45], or the hitherto smallest αvβ6-integrin specific RGD, the cyclic pentapeptide SDM17 by Marinelli and collegues [46,47].

## 5. The Future of RGD Is Bright

With this in mind, it is worth taking a look beyond radiopharmaceuticals. It is not difficult to anticipate that a cancer-cell specific molecular target such as αvβ6-integrin is also highly attractive for development of non-radioactive imaging probes and particularly for homing of therapeutic agents. The latter applies even more as any form of therapeutic agent greatly benefits from the clinical availability of a companion diagnostic, i.e., an established diagnostic procedure for patient stratification, such as the αvβ6-integrin PET imaging modality shown in Figure 1.

The most exciting prospect is, arguably, the fact that αvβ6-integrin is apparently a valuable target for efficient intracellular drug delivery [48]. Although it seems rather obvious to exploit the internalization of a cell surface receptor to transfer cytotoxic compounds into cells, it is surprising to see that this approach apparently works robustly with fundamentally different αvβ6-integrin selective RGD peptides. Marshall and colleagues recently designed a peptide-drug conjugate (PDC) based on their classic A20FMDV peptide, and successfully wiped out pancreatic cancer cells in vitro and in animal models [49]. Their statement: “[…] it is clear that specific targeting αvβ6 in humans is now a practical possibility and should become a platform for development of improved therapies for the effective treatment of pancreatic and hopefully many other types of cancer” [49] is bolstered by recent works of the Sutcliffe group, who used another PDC based on their linear peptide with similar success in vitro and in vivo [50], and by Kossatz and colleagues who decorated one of the cyclic nonapeptides developed by Maltsev et al. [43] with the cytotoxic Fenton catalyst ferrocene and selectively killed αvβ6-positive tumor cells with the conjugate [51]. The αvβ6-integrin-mediated, selective transmembrane drug delivery into cancer cells appears to be a truly robust machinery that tolerates a wide range of substrates, which nurtures the hope that pertinent research eventually yields novel, highly effective anticancer drugs with few side effects that are so desperately needed by many cancer patients.

These examples, however, still represent only part of a much bigger picture. There are many more approaches to exploit integrins for therapy [32]. Not all of these involve RGD peptides, above all, because non-RGD-binding integrins (not covered herein) are also valid therapeutic targets. On the other hand, there are many non-peptidic ligands for any of the RGD-recognizing integrins, for example, a small-molecule ligand with high αvβ6-integrin affinity that was recently tested in a clinical study for treatment of lung fibrosis by means of inhalation [52]. All in all, research on drugs and probes targeting RGD-binding integrins does not necessarily have to involve RGD peptides, but much of the related future development will certainly continue to rely on them.

## 6. Epilogue

At times, it is difficult even for experts to predict the future of a particular technology, and by whom it will be most appreciated. Consider, for example, the beauty industry’s use of lasers to remove unwanted hair and wrinkles, which was certainly not foreseen by Maiman and colleagues in 1960 but nowadays helps many people with absolutely no knowledge about coherent optical radiation to become a little more beautiful—and yet may have utterly failed to increase the physical attractiveness of some contemporary laser experts despite of their strong belief it would do so. Some parallels to RGD can be discovered here as well. Just as not every problem can be solved with lasers despite of their broad technological range, not all future developments related to RGD-recognizing integrins and their ligands will rely on RGD peptides despite their ubiquity. Current research trends nonetheless strongly suggest that RGD will remain an important tool in future molecular medicine and life science. Newly discovered roles of integrins in biochemical pathways might open new avenues for the use of certain specific RGD peptides even in areas that previously had no real ties to RGD, such as cancer immunotherapy [53,54]. In view of the ever-expanding knowledge of the complexity of integrin functions [13], it is, arguably, quite probable that future RGD-related research will focus less on angiogenesis and αvβ3-integrin, which has been already exhaustively addressed in the past decade. Those who lament on this loss might be consoled by the prospect that maybe there will be a revival in the future, around the time when spaceships are equipped with lasers.

## Data Availability

Data sharing not applicable.
